# Inflammatory Markers and Their Association With Insulin Resistance in Indian Children and Young Adults With Type 1 Diabetes

**DOI:** 10.7759/cureus.92669

**Published:** 2025-09-18

**Authors:** Madhura Karguppikar, Anuradha Khadilkar, Shruti Mondkar, Aboli Bhalerao, Sonali Wagle, Vaman Khadilkar

**Affiliations:** 1 Growth and Pediatric Endocrinology, Smt. Kashibai Navale (SKN) Medical College, Pune, IND; 2 Growth and Pediatric Endocrinology, Hirabai Cowasji Jehangir Medical Research Institute, Pune, IND; 3 Interdisciplinary School of Health Sciences, Savitribai Phule University, Pune, IND

**Keywords:** indian, inflammatory markers, insulin resistance, pediatric, type 1 diabetes

## Abstract

Introduction: Long-term hyperglycemia in type 1 diabetes (T1D) leads to inflammation, oxidative stress, and endothelial damage. Chronic degeneration may lead to higher levels of inflammatory markers. The present study aimed to evaluate inflammatory markers and their association with insulin resistance in Indian children and youth with T1D. It further explores the predictors of inflammatory markers in them.

Methods: 194 children and youth (11.6-17.5 years) with T1D for at least two years were included in this cross-sectional study. Standard questionnaires and protocols were used to obtain demographic data and laboratory findings. High-sensitivity C-reactive protein (hs-CRP), interleukin-6 (IL-6), and tumor necrosis factor α (TNF-α) were analyzed as inflammatory markers. Insulin sensitivity was computed using parameters required for SEARCH.

Results: 14.4% were found to have insulin resistance. Those with insulin resistance were found to have significantly lower glycemic control (HbA1c) and lean body mass (LBM) Z-score. Also, those with insulin resistance had higher insulin requirement, altered albumin-to-creatinine ratio (ACR), low-density lipoprotein (LDL) cholesterol, and hypertension. hs-CRP, IL-6, and TNF-α were higher in those with higher insulin resistance. By linear regression, higher hs-CRP, ACR, LDL, HbA1c, and fat percentage were important predictors of insulin resistance.

Conclusions: T1D with insulin resistance is associated with higher inflammatory markers and subsequent microvascular and macrovascular complications. Early screening and timely intervention are required to abate disease progression and hypertension and avoid end-stage kidney disease.

## Introduction

Long-standing, poorly controlled diabetes is associated with end-organ damage [1]. Cardiovascular (CV) health is at risk in them, and regular assessment of lipid parameters and lipoproteins is recommended. These traditional factors do not account for the overall risk of CV disease (CVD). Non-traditional CVD risk factors may be classified as coagulation-related, metabolism-related, and inflammation-related. These are explored in adults with type 2 diabetes (T2D), and they are shown to be associated with poor CVD outcomes [2].

Given the global increase in obesity, individuals with type 1 diabetes (T1D) are also at a higher risk of metabolic syndrome. In addition to the insulin deficiency state, they also tend to have insulin resistance. Its etiology is multifactorial and results in "double diabetes" (DD). Indians are at a higher risk given the predisposition to central obesity and metabolic syndrome. While insulin resistance is cumbersome to quantify in those on insulin therapy, there are equations that help in the estimation of insulin resistance in those with endogenous insulin deficiency. The estimated glucose disposal rate (eGDR) is a marker showing good correlation with the euglycemic-hyperinsulinemic clamp (EHC) test (gold standard for assessing insulin resistance) and has been validated for use in individuals with T1D. The SEARCH equation takes into account the waist circumference (WC), HbA1c, and serum triglyceride levels to estimate insulin resistance [3].

Weight gain and adiposity are central factors known to be associated with insulin resistance. Poor glycemic control leads to increased insulin requirement, which in turn leads to insulin-induced weight gain. This is a vicious cycle, which leads to insulin resistance. While intensive insulin therapy and good glycemic control have shown decreased frequency of microangiopathic complications, this has not reduced macrovascular complications due to CV risk factors [3]. The present view of atherosclerosis pathogenesis is based on a response to injury model. Here, the vascular endothelium is injured by blood flow abnormalities, which are exacerbated by the effects of traditional and other risk factors. The formation and development of plaque are due to cellular and molecular responses to inflammatory action (documented as an increase in inflammatory markers) [4]. While these markers are most pronounced during acute CV events, they may also be involved in the latent stages of disease progression.

Taken together, a better understanding of the risk of developing CVD, considering the additional danger due to the presence of insulin resistance, in T1D is required. Furthermore, there are very limited data regarding this in children and young adults from the Indian subcontinent. The present study thus aimed to evaluate inflammatory markers and their association with insulin resistance in Indian children and youth with T1D. It further explores the predictors of inflammatory markers in these children and young adults.

## Materials and methods

Subjects and study design

One hundred and ninety-four children with T1D (duration of diabetes more than two years), along with their parents who were receiving care at the pediatric endocrine unit at a tertiary care hospital in Pune, India, were approached to take part in this cross-sectional, observational study. The pediatric endocrine unit in our center runs a multidisciplinary clinic for underprivileged children and young adults with T1D. The venture supports medical care essentials like glucometers, lancets, strips, insulin, other medications, medical consultations, psychologists' and nutritionists' opinions, social workers, etc. Since monitoring for complications of T1D is recommended after two years of disease duration, those with diabetes duration less than two years were not included in the study [5]. Children with other major illnesses or comorbidities (like celiac disease, untreated hypothyroidism, and/or polyendocrinopathy) were excluded from the study. Post hoc power calculation (G*Power, Version 3.1, Heinrich-Heine-Universität Düsseldorf, Düsseldorf, Germany), at a significance level of 0.05, yielded a power of 0.8 for which a sample size of 194 was adequate.

This study was conducted at the Sweetlings Program of Hirabai Cowasji Jehangir Medical Research Institute at Jehangir Hospital, Pune, India, between May 2024 and October 2024. Ethics approval was obtained from the Biomedical and Health Research Ethics Committee of Jehangir Clinical Development Centre Pvt. Ltd. (approval number: EC/NEW/INST/2023/MH/0236). Parents provided written informed consent, and children gave verbal assent for the study.

Clinical history and examination

Standardized questionnaires were used by physicians to collect data on demographics and medical history. This was verified from hospital medical records after data collection from parents. The Tanner staging for sexual maturity was performed by a pediatric endocrinologist after verbal consent in the presence of an attendant. Blood pressure (BP) was measured using a mercury sphygmomanometer, with an appropriately sized cuff. After a rest of at least five minutes, BP was recorded in the sitting or supine position, and the cubital fossa was supported at the heart level. BP was measured again after 10 minutes and confirmed by another examiner. In case of a high reading, systolic BP (SBP) and/or diastolic BP (DBP) >95th percentile was classified as hypertension in subjects less than 13 years [6]. In subjects over 13 years of age, BP readings of SBP >130 mmHg and DBP >80 mmHg were defined as hypertension [7].

Anthropometry

Standing height using a portable stadiometer (Leicester Height Meter, Child Growth Foundation, Newcastle upon Tyne, England) was measured to the nearest millimeter, and weight was measured using an electronic weighing scale to the nearest 100 grams. Body mass index (BMI) was computed by dividing weight in kilograms by height in meters squared. The height, weight, and BMI were converted to Z-scores using Indian references [8]. Using the above reference standards and BMI Z-scores, we classified them as overweight and obese. WC and hip circumference were measured using the World Health Organization (WHO) guide to physical measurements [9].

Biochemical measurements

Blood samples (5 ml) were collected between 7 and 9 am in a fasting state by a trained pediatric phlebotomist. The fasting blood samples were then assessed for lipid profile (total cholesterol, triglycerides, and high-density lipoprotein cholesterol (HDL-C)) using the enzymatic method, and low-density lipoprotein cholesterol (LDL-C) concentrations were calculated using the Friedewald formula [10]. Subjects were classified to have dyslipidemia if one or more of the following lipid parameters were abnormal: LDL-C >100 mg/dl (>2.6 mmol/L), HDL-C <40 mg/dl (<1.1 mmol/L), total cholesterol >200 mg/dl (>5.2 mmol/L), and triglycerides >130 mg/dl (>1.5 mmol/L) in children aged >10 years and 100-130 mg/dl (1.1-1.5 mmol/L) in children <10 years [11]. HbA1c was measured by high-performance liquid chromatography (HPLC) (Bio-Rad, Germany). Thyroid-stimulating hormone (TSH) concentrations were measured by chemiluminescent microparticle immunoassay (CMIA).

Inflammatory markers

hs-CRP was assessed using the turbidimetry method, IL-6 using electrochemiluminescence immunoassay, and TNF-α using enzyme-linked immunosorbent assay.

A sterile container was used to collect the first voided, morning urine sample. The sample was processed to determine urine microalbumin (by radioimmunoassay) and urine creatinine (Jaffe's method). Urine albumin-to-creatinine ratio (ACR) was computed using a ratio of urine albumin to urine creatinine. Samples were not taken during menstrual periods, during instances of fever, following intense exercise, or during significant hyperglycemia. Albuminuria was defined as ACR 2.5-25 mg/mmol or 30-300 mg/g (spot urine) in males and 3.5-25 mg/mmol or 42-300 mg/g in females (due to lower creatinine excretion) [12].

eGDR was computed for all patients using the following formula: $\text{Exp (4.64725-0.02032 (waist circumference)-0.09779 (HbA1c)-0.00235 (triglycerides))}$. As a cut‐off, 5.485 mg/kg/min was found to have the highest sensitivity and specificity in a previous study by our group; this was considered the criterion for the conversion of the insulin sensitivity variable into categorical data for analysis [13].

Carotid intima-media thickness (cIMT)

cIMT was measured by a blinded, single radiologist using the ultrasound B-mode. Far-wall cIMT was assessed from standard magnified images of the far (posterior) wall of the common carotid artery, immediately proximal to the carotid bulb. The maximum distance between the media-adventitia interface and the lumen-intima interface was recorded.

Body composition

After a minimum of three hours of fasting and voiding before measurements, body composition was assessed using a bioelectrical impedance analyzer (BIA) (Tanita Model BC-420MA, Tokyo, Japan). BIA measures body composition as fat percentage, fat mass, fat-free mass (FFM), lean body mass (LBM), total body water, and bone mineral amount included in the entire bone (bone mass) by measuring bioelectrical impedance in the standing position. Indian reference data were used to compute Z-scores for body fat percentage and lean mass percentage [14].

Statistical analysis

All statistical analyses were carried out using IBM SPSS Statistics for Windows, Version 29.0 (IBM Corp., Armonk, New York, United States). All variables were tested for normality before performing statistical analysis using the Shapiro-Wilk test. Variables, which were normally distributed, were presented as mean (SD), whereas non-normalized data were presented as median (25th percentile, 75th percentile). Student's t-test was used to check the mean differences between the two groups. The Mann-Whitney U test was used to check the median differences between the groups. To find the predictors of eGDR, univariate linear regression was performed with age, duration of diabetes, height Z-score, weight Z-score, BMI Z-score, WC, HbA1c, CRP, dose of insulin (IU) per kg body weight per day, cIMT, femoral intima-media thickness, IL-6, TNF-α, ACR, cholesterol, HDL, LDL, triglyceride, fat percentage, fat percentage Z-score, LBM percentage, LBM Z-score, and FFM. Variables that showed significance in univariate regression were selected for multivariate linear regression. For testing relationships between dichotomous-dependent variables and continuous predictors, univariate linear regression analysis was carried out. P-values <0.05 were considered statistically significant.

## Results

Two hundred and eighty-eight patients with T1D were approached to take part in this study. Of these, 234 met the inclusion criteria. Of the 54 who were not included, 50 had a disease duration of less than two years, and four had poorly controlled/untreated hypothyroidism. The study was carried out on 194 participants: 47.4% (92) males and 52.6% (102) females. The minimum and maximum ages of the participants involved in the study were 11.6 years and 17.5 years, respectively. The mean HbA1c was 10.1±2% (87 mmol/L).

All children were on a basal bolus regimen. The mean total insulin requirement in our cohort was 1.1±0.4 units/kilogram/day. Patient demographics and laboratory findings have been illustrated in Table 1.

**Table 1:** Demographic data of the children/youth with T1D The data have been represented as N, %, mean±SD, and median (25th to 75th percentile). P1-value is calculated by non-parametric test, expressed as median±interquartile range. P2-value is calculated by t-test, expressed as mean±SD. T1D: type 1 diabetes; BMI: body mass index; IMT: intima-media thickness; hs-CRP: high-sensitivity C-reactive protein; IL-6: interleukin 6; TNF-α: tumor necrosis factor α; LDL: low-density lipoprotein; HDL: high-density lipoprotein; eGDR: estimated glucose disposal rate; LBM: lean body mass

	Male (n=92)	Female (n=102)	Z-value	P1-value	t-value	P2-value
Age (years)	15.16 (12.07, 17.47)	14.11 (11.57, 17.55)	-0.511	0.609	0.267	0.790
Duration of diabetes	6.76 (3.7, 9.74)	6.23 (3.77, 9.74)	-0.047	0.962	-0.856	0.393
Height Z-score	-0.96 (-1.57, -0.02)	-0.76 (-1.53, 0.02)	0.851	0.395	-0.888	0.376
Weight Z-score	-0.61 (-1.48, -0.03)	-0.58 (-1.24, 0.18)	1.097	0.273	-0.773	0.441
BMI Z-score	-0.48 (-1.12, 0.08)	-0.35 (-0.9, 0.35)	1.195	0.232	-0.812	0.418
Waist circumference (cm)	69.5 (61, 78)	68.25 (63.5, 74.13)	-0.289	0.772	1.934	0.651
HbA1c	9.75 (8.93, 11.48)	9.65 (8.5, 10.93)	-2.014	0.139	-0.453	0.055
Dose of insulin (IU) per kg body weight per day	1.11 (0.89, 1.31)	1.14 (0.87, 1.40)	-0.367	0.578	0.623	0.357
Hypertension	84	97	-	-	-	-
n (%)	6.0 (7.1%)	4.0 (4.1%)	0.786	0.375	-	-
Carotid IMT	0.35 (0.35, 0.4)	0.35 (0.3, 0.4)	-2.014	0.044	1.915	0.057
Femoral IMT	0.3 (0.25, 0.35)	0.3 (0.25, 0.3)	-0.436	0.663	0.86	0.391
hs-CRP	1.03 (0.81, 2.1)	2.4 (1.27, 4.37)	5.314	<0.001	-5.288	<0.001
IL-6	1.5 (1.5, 2.1)	1.61 (1.5, 2.74)	0.906	0.365	-0.654	0.515
TNF-α	12.4 (8.8, 18.13)	11.27 (8.25, 20.63)	-0.491	0.624	0.572	0.569
Albumin-to-creatinine ratio	15.95 (8.08, 23.34)	19.25 (10.51, 27.64)	2.405	0.016	-2.152	0.033
Cholesterol (mg/dl)	152.5 (137, 170.75)	163 (142, 186.25)	2.302	0.021	-2.439	0.016
HDL (mg/dl)	40 (34, 44)	39 (36, 47)	0.793	0.427	-1.095	0.275
LDL (mg/dl)	96.1 (83.46, 110.25)	104.3 (87.15, 125.1)	2.172	0.03	-2.114	0.036
Triglyceride (mg/dl)	83.5 (61.5, 91)	85 (60.5, 97.25)	0.845	0.398	-1.168	0.244
Fat percentage	13.3 (8.2, 16.9)	23.6 (18.8, 29)	8.312	<0.001	-9.353	<0.001
Fat percentage Z-score	-0.29 (-1.03, 0.24)	-0.12 (-0.66, 0.4)	1.853	0.064	-1.848	0.066
LBM percentage	85.82 (79.93, 90.61)	75.37 (68.68, 79.99)	-7.966	<0.001	8.852	<0.001
LBM Z-score	0.85 (0.1, 1.57)	0.57 (-0.17, 1.19)	-1.837	0.066	1.88	0.062
FFM (kg)	38.1 (28.2, 45.8)	29.2 (25.35, 33.1)	-5.087	<0.001	5.128	<0.001
eGDR SEARCH	7.99 (6.41, 9.94)	8.17 (6.61, 9.97)	0.607	0.544	-0.833	0.406

**Figure 1:**
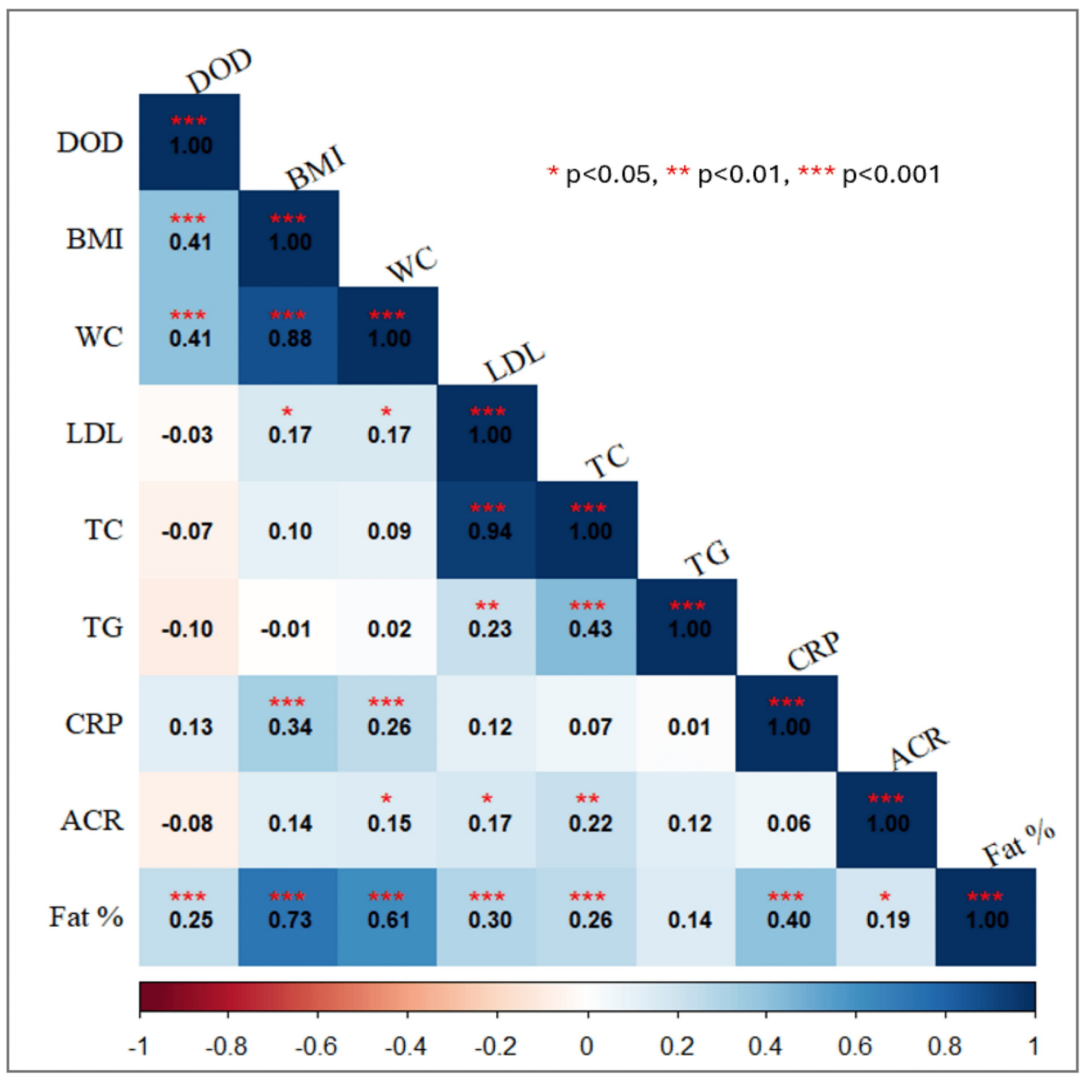
Correlogram showing the correlation between parameters DOD: duration of diabetes; BMI: body mass index; WC: waist circumference; LDL: low-density lipoprotein; TC: total cholesterol; TG: triglycerides; CRP: C-reactive protein; ACR: urinary albumin-to-creatinine ratio; Fat %: body fat percentage

Using the SEARCH equation, 28 patients were found to have insulin resistance (eGDR <5.486). Insulin resistance was noted to be higher in those with higher age (p<0.001) and greater disease duration (p<0.05). Also, those with insulin resistance had higher HbA1c (p<0.001), total cholesterol (p<0.001), LDL-C (p<0.001), and urinary ACR (p<0.01). In the T1D group, 17.9% (n=5) of children were noted to be overweight with insulin resistance as compared to 10.8% (n=18) without insulin resistance. Similarly, 21.4% (n=6) were noted to be obese in the insulin resistance group as compared to 2.4% (n=4) in the group without insulin resistance. Overweight and obesity were noted to be significantly higher in those with insulin resistance. Body composition revealed those with insulin resistance to have a higher fat percentage Z-score (p<0.01) and a lower LBM Z-score (p<0.006). Hypertension was significantly higher in those with insulin resistance (11.1%) as compared to those without insulin resistance (4.5%). Interestingly, hs-CRP (p<0.05) and IL-6 (p<0.05) were noted to be significantly higher in those with insulin resistance. Though TNF-α too was higher in those with insulin resistance, it did not reach statistical significance. Comparison of parameters based on insulin resistance is depicted in Table 2. A correlogram showing the correlation between variables is illustrated in Figure 1.

**Table 2:** Comparison of parameters as per eGDR eGDR value (calculated as per the SEARCH study) of <5.485 mg/kg/min taken as a cut-off for insulin resistance. Values are in n(%) or median (LQ, UQ). P1-value is calculated by non-parametric test. P2-value is calculated by t-test. BMI: body mass index; IMT: intima-media thickness; hs-CRP: high-sensitivity C-reactive protein; IL-6: interleukin 6; TNF-α: tumor necrosis factor α; LDL: low-density lipoprotein; HDL: high-density lipoprotein; eGDR: estimated glucose disposal rate; LBM: lean body mass; FFM: fat-free mass

Parameters	eGDR (SEARCH) ≤5.486	eGDR (SEARCH) >5.486	Z-value	P1-value	t-value	P2-value
	(n=28)	(n=166)				
Male	16.0 (57.1%)	76.0 (45.8%)	1.24	0.265	-	-
Age (years)	16.74 (14.35, 18.16)	14.02 (11.11, 17.01)	-3.193	0.001	3.077	0.002
Duration of diabetes	8.55 (4.15, 11.39)	6.13 (3.61, 9.62)	-1.943	0.052	1.912	0.057
Height Z-score	-0.95 (-1.65, -0.08)	-0.83 (-1.56, 0)	0.884	0.377	-1.24	0.216
Weight Z-score	-0.44 (-1.09, 0.47)	-0.61 (-1.39, 0)	-1.237	0.216	1.596	0.112
BMI Z-score	-0.18 (-0.73, 0.96)	-0.42 (-1.01, 0.12)	-2.158	0.031	2.755	0.006
Waist circumference (cm)	77.75 (72.13, 88.25)	67.5 (61, 73.5)	-5.012	<0.001	9.771	<0.001
HbA1c	13.25 (11.2, 14.43)	9.4 (8.5, 10.63)	-6.95	<0.001	4.481	<0.001
Dose of insulin (IU) per kg body weight per day	1.11 (0.81, 1.45)	1.12 (0.88, 1.34)	-3.457	0.977	3.256	0.673
Carotid IMT	0.4 (0.35, 0.4)	0.35 (0.3, 0.4)	-1.565	0.118	1.395	0.165
Femoral IMT	0.3 (0.25, 0.35)	0.3 (0.25, 0.3)	-0.275	0.784	-0.085	0.932
hs-CRP	2.65 (1.22, 4.28)	1.45 (0.91, 3.06)	-2.02	0.043	2.331	0.021
IL-6	2.65 (1.6, 3.76)	1.5 (1.5, 2.22)	-1.893	0.058	0.969	0.336
TNF-α	12.45 (5.15, 20.98)	11.54 (8.55, 18.2)	-0.125	0.9	0.028	0.977
Albumin-to-creatinine ratio	23.43 (13.12, 53.73)	16.27 (8.63, 25.68)	-2.483	0.013	2.75	0.007
Cholesterol (mg/dl)	174.5 (158.25, 193.25)	153.5 (137, 172.25)	-3.495	<0.001	3.587	<0.001
HDL (mg/dl)	39 (33.25, 44)	40 (36, 45)	0.749	0.454	-0.866	0.387
LDL (mg/dl)	114.3 (100.05, 133.25)	96.55 (83.4, 115)	-3.262	0.001	3.224	0.001
Triglyceride (mg/dl)	90 (77.5, 144.5)	82.5 (55.75, 93)	-3.337	<0.001	4.489	<0.001
Fat percentage	23.7 (16.05, 31.8)	17.85 (12.3, 23.7)	-2.607	0.009	2.66	0.009
Fat percentage Z-score	0.18 (-0.55, 1.09)	-0.2 (-0.89, 0.23)	-2.904	0.004	3.59	<0.001
LBM percentage	73.95 (66.52, 83.25)	80.21 (74.23, 87.01)	2.613	0.009	-2.992	0.003
LBM Z score	0.38 (-0.97, 0.92)	0.72 (0.1, 1.44)	2.744	0.006	-3.448	<0.001
FFM (kg)	39.45 (29.25, 46.75)	31.2 (25.13, 38.05)	-3.313	0.002	3.306	0.001

Univariate linear regression showed that lower age, disease duration, weight Z-score, WC, HbA1c, hs-CRP, IL-6, cIMT, total cholesterol, LDL, triglycerides, and fat percentage have higher eGDR associated with lower insulin resistance (Table 3). Furthermore, multiple linear regression showed lower WC, HbA1c, and triglycerides to be predictors of high eGDR.

**Table 3:** Univariate linear regression to determine predictors of eGDR (p<0.05) BMI: body mass index; IMT: intima-media thickness; IL-6: interleukin 6; TNF-α: tumor necrosis factor α; LDL: low-density lipoprotein; HDL: high-density lipoprotein; eGDR: estimated glucose disposal rate; LBM: lean body mass; FFM:

	All		Male		Female	
	Std. ß	P-value	Std. ß	P-value	Std. ß	P-value
Age (years)	-0.485	<0.001	-0.594	<0.001	-0.363	<0.001
Duration of diabetes	-0.259	<0.001	-0.35	<0.001	-0.141	0.165
Height Z-score	0.016	0.83	0.052	0.622	-0.019	0.847
Weight Z-score	-0.225	0.002	-0.193	0.066	-0.269	0.006
BMI Z-score	-0.299	<0.001	-0.246	0.018	-0.365	<0.001
Waist circumference (cm)	-0.642	<0.001	-0.658	<0.001	-0.675	<0.001
HbA1c	-0.704	<0.001	-0.712	<0.001	-0.697	<0.001
CRP	-0.204	0.004	-0.207	0.048	-0.281	0.004
Dose of insulin (IU) per kg body weight per day	-0.110	0.131	-0.227	0.031	-0.099	0.328
Carotid IMT	-0.157	0.029	-0.174	0.098	-0.130	0.194
Femoral IMT	0.016	0.823	0.048	0.647	-0.036	0.716
IL-6	-0.279	0.017	-0.268	0.119	-0.275	0.095
TNF-α	-0.066	0.58	-0.221	0.21	0.045	0.789
Albumin-to-creatinine ratio	-0.237	<0.001	-0.211	0.043	-0.290	0.003
Cholesterol (mg/dl)	-0.311	<0.001	-0.178	0.09	-0.479	<0.001
HDL (mg/dl)	0.179	0.012	0.208	0.047	0.137	0.169
LDL (mg/dl)	-0.311	<0.001	-0.222	0.033	-0.434	<0.001
Triglyceride (mg/dl)	-0.382	<0.001	-0.196	0.061	-0.565	<0.001
Fat percentage	-0.347	<0.001	-0.457	<0.001	-0.553	<0.001
Fat percentage Z-score	-0.411	<0.001	-0.416	<0.001	-0.443	<0.001
LBM percentage	0.36	<0.001	0.464	<0.001	0.533	<0.001
LBM Z-score	0.406	<0.001	0.402	<0.001	0.446	<0.001
FFM (kg)	-0.5	<0.001	-0.577	<0.001	-0.403	<0.001

## Discussion

Our cross-sectional study showed that 28 (14.4%) underprivileged children and youth with T1D have insulin resistance. Children with insulin resistance were found to have significantly greater disease duration, significantly higher WC, HbA1c, total cholesterol, triglycerides, ACR, hs-CRP, IL-6, and fat percentage Z-score, and lower LBM Z-score.

T1D is known to be associated with multiple microvascular (nephropathy, retinopathy, neuropathy) and macrovascular (CVD and stroke) complications. Poor glycemic control is associated with the early onset of these complications. Similarly, obesity and the subsequent development of metabolic syndrome and T2D are known to be associated with similar complications. While T1D remains the leading cause of diabetes in the pediatric age group, recent trends in changes in lifestyle patterns have led to a rise in cases of pediatric obesity and subsequently T2D [15]. Asian Indians are at a higher risk of insulin resistance from early infancy, and insulin resistance syndrome has been encountered in children as young as eight years old [16].

Due to changing trends, there is a higher DD seen in recent years [17]. The entity encompasses the autoimmune damage of T1D combined with the metabolic damage of T2D. Emerging insulin resistance and its clinical markers may also be clinically evident in individuals with T1D. Traditionally, individuals with T1D have a lean body habitus with normal body weight. With changes in global trends, individuals with T1D may also show signs of insulin resistance, like overweight/obesity, acanthosis nigricans, and irregular cycles with polycystic ovaries (in girls) [18]. Poor glycemic control leads to increased insulin requirement, which in turn leads to insulin-induced weight gain. This is a vicious cycle that leads to insulin resistance.

Our study reported that 14.4% (n=28) of children and young adults have insulin resistance. Pozzilli et al. reported the prevalence of DD to be 4.96% in Caucasian individuals [19]. Lawrence et al. reported that in a multicenter study with a mean of 3.47 million youth, for each prevalence year from six areas in the United States, the estimated prevalence of T1D among those under 19 years increased significantly, from 1.48 to 2.15 per 1000 youths, and the estimated prevalence of T2D among those aged 10-19 years increased from 0.34 to 0.67 per 1000 youths [20]. Globally, there is a paucity of data on the incidence of insulin resistance in children with T1D. The primary reason for this could be the practical difficulties in estimating insulin resistance in individuals on exogenous insulin. While the Homeostasis Model Assessment of Insulin Resistance (HOMA-IR) is widely used in estimating insulin sensitivity, its calculation requires fasting insulin and fasting glucose. HOMA-IR cannot be used as an indicator of insulin sensitivity/insulin resistance in T1D patients as they are on exogenous insulin. To combat this, several equations have been proposed and validated against data from EHC tests (the gold standard) to estimate whole-body insulin sensitivity [21]. Other equations developed to estimate insulin resistance in patients with T1D include the following: (1) the Epidemiology of Diabetes Complications (EDC) equation that was initially developed in the Pittsburgh EDC Study which considers glycemic control, waist-to-hip ratio (WHR), and BP, (2) the estimated insulin sensitivity (eIS)-Coronary Artery Calcification in Type 1 Diabetes (CACTI) equation (using WC, insulin dose, triglycerides, and DBP), and (3) the SEARCH study's eIS score using WC, HbA1c, and triglycerides.

A study by our group comparing insulin sensitivity indices for the detection of DD in Indian adolescents with T1D found the SEARCH equation to have maximum sensitivity and specificity in determining metabolic syndrome. It proposes that insulin sensitivity determined by the SEARCH equation may be used in routine clinical practice to detect DD in Indian adolescents with T1D at risk of developing metabolic as well as microvascular complications [13]. Our group's research found the cut-off value of 5.485 mg/kg/min to have the highest sensitivity and specificity in identifying metabolic syndrome, and we have used the same cut-off in this study [13].

Individuals of Southeast Asian descent tend to have higher body fat (particularly abdominal fat) as compared to other ancestries [22]. This puts them at a higher risk of insulin resistance at a lower BMI. Indian neonates preserve body fat even in the intrauterine life; this phenotype persists in adulthood, resulting in significant differences in body composition [23]. Truncal body fat pattern, abdominal obesity, increased body fat, and insulin resistance were noted to be higher in post-pubertal children in India [24]. Another study reported that 64% of obese adolescents in India have fasting hyperinsulinemia, suggesting insulin resistance [25]. Our study showed that children with insulin resistance have significantly higher WC and BMI. Davis et al. noted an increase in body fat at one year after diagnosis and treatment with insulin in children with T1D. The same study also reported girls to have higher HbA1c and cholesterol levels despite being on higher doses of insulin, suggesting insulin resistance and increased CV risk [26]. Our study reported significantly higher fat percentage and lower LBM in girls. We also reported that children with insulin resistance have a significantly higher fat percentage and Z-score and a lower LBM and Z-score. These findings suggest a relationship between higher fat percentage, lower LBM, and insulin resistance. Hypertension has been reported in 6-16% children with T1D in the literature. It is a modifiable CV risk factor that often goes undiagnosed and undertreated [27]. Our study reported significantly higher hypertension in those with insulin resistance.

Both T1D and insulin resistance lead to oxidative stress and subclinical levels of inflammation, which cause slow, persistent damage to the endothelial surfaces. T1D is associated with inflammatory processes throughout its course. The first bout of inflammation occurs at the onset of the disease through the lymphocyte-mediated destruction of beta cells of the pancreas. Once the disease sets in, this acute inflammation subsides and is followed by chronic inflammation, which is intermittently exacerbated by episodes of hyperglycemia. In children with poor glycemic control, repeated hyperglycemia leads to the inability to fully correct from pro-inflammatory effects, and this results in damaging vascular effects. A study by Rosa et al. demonstrated an increase in IL-6, TNF-α, and other cytokines in adolescents with T1D. Various other studies have shown overweight/obesity to be associated with increased levels of inflammatory markers and early atherosclerosis [28].

The role of insulin resistance in the pathogenesis of dyslipidemia is widely studied in both insulin-dependent and insulin-independent diabetes. Several studies suggest that dyslipidemia is a factor in causing insulin resistance. High levels of very-low-density lipoprotein (VLDL), HDL, and apolipoprotein A1 are known to be associated with insulin resistance [29]. Recent studies suggest the possibility of high concentrations of triglyceride-rich VLDL particles impairing insulin action by inhibiting insulin binding to its receptor [30]. Insulin resistance may therefore be secondary to primary dyslipidemia. Hence, insulin resistance and dyslipidemia seem to have a cause-and-effect relationship. Increased insulin resistance leads to dyslipidemia, and dyslipidemia further leads to the worsening of insulin resistance, amounting to a vicious cycle. Our study shows dyslipidemia to be a significant predictor of insulin resistance.

The strength of our study is that ours is one of the very few Asian studies to report the association of inflammatory markers with insulin resistance and its predictors. The limitations of our study include that it is a single-center study. Comparison between baseline and current characteristics was not possible as biochemical investigations and anthropometric data were not available on all patients at the time of diagnosis. Additionally, ours is a study on underprivileged children in whom the control of diabetes was not optimal. Hence, results may not be generalizable to children from other socioeconomic strata. Further, while our findings demonstrate significant associations between inflammatory markers and insulin resistance in children with T1D, causality cannot be inferred. It remains unclear whether inflammation contributes to the development of insulin resistance or whether insulin resistance itself promotes chronic low-grade inflammation. The temporal sequence of metabolic changes, including whether elevated inflammatory markers predict future complications, cannot be assessed in the present design. Longitudinal studies are thus needed to clarify these causal pathways and to determine whether baseline inflammatory profiles in children with T1D can serve as predictors of long-term CV and renal outcomes.

## Conclusions

T1D with insulin resistance results in the double burden of inflammation and accelerated damage. Further, Indian children with T1D are at an increased risk of insulin resistance and inflammatory changes owing to their ethnicity and higher propensity to develop insulin resistance. The importance of lifestyle changes and close monitoring needs to be emphasized to prevent the onset of insulin resistance. Routine screening of cardiac health markers (including inflammatory markers) may be considered by clinicians, especially in individuals with T1D and at risk of metabolic syndrome. Further large-scale, longitudinal studies are required to assess the use of eGDR and inflammatory markers in assessing insulin resistance and complications to aid early diagnosis and intervention.
